# Supplementary material for: Increased vaccine sensitivity of an emerging SARS-CoV-2 variant
Source: Nat Commun. 2023 Jun 29;14:3854. doi: 10.1038/s41467-023-39567-2 (PMC10310822; doi:10.1038/s41467-023-39567-2)
Supplement: Supplementary file 1 — Supplementary Information [file 41467_2023_39567_MOESM1_ESM.pdf]

Increased vaccine sensitivity of an emerging SARS-CoV-2 variant: supporting information

Joseph A. Lewnard[1,2,3,*], Vennis Hong[4], Jeniffer S. Kim[4], Sally F. Shaw[4], Bruno Lewin[4,5], Harpreet Takhar[4], Marc Lipsitch[6], Sara Y. Tartof[4,7,*]

1. Division of Epidemiology, School of Public Health, University of California, Berkeley, Berkeley, California 94720, United States
2. Division of Infectious Diseases & Vaccinology, School of Public Health, University of California, Berkeley, Berkeley, California 94720, United States
3. Center for Computational Biology, College of Engineering, University of California, Berkeley, Berkeley, California 94720, United States
4. Department of Research & Evaluation, Kaiser Permanente Southern California, Pasadena, California 91101, United States
5. Department of Clinical Science, Kaiser Permanente Bernard J. Tyson School of Medicine, Pasadena, California 91101, United States
6. COVID-19 Response Team, Centers for Disease Control and Prevention, Atlanta, Georgia, 30329, United States
7. Department of Health Systems Science, Kaiser Permanente Bernard J. Tyson School of Medicine, Pasadena, California 91101, United States

* Addresses for correspondence:

Joseph A. Lewnard
2121 Berkeley Way
Berkeley, California 94720
jLewnard@berkeley.edu
510-664-4050

Sara Y. Tartof
100 South Los Robles
Pasadena, California 91101
Sara.Y.Tartof@kp.org
626-564-3001

Contents of this supplement

Item	Title	Page
Table S1	History of vaccination and natural infection among cases with S-gene target failure or S-gene detection.	2
Table S2	Association of S-gene detection with prior vaccination before or after natural SARS-CoV-2 infection.	3
Table S3	Association of S-gene detection with prior documented infection and COVID-19 vaccination, estimated via models defining continuous functions for calendar time.	4
Table S4	Association of S-gene detection with prior COVID-19 vaccination status, according to timing of most recent vaccination.	5
Table S5	Association of S-gene detection with prior COVID-19 vaccination status, according to vaccine type.	6
Table S6	Association of S-gene detection with most recent vaccine product received.	7
Table S7	Vaccine dose sequences among cases with S-gene target failure or S-gene detection.	8
Table S8	Association of S-gene detection with prior documented infections during periods with differing predominant circulating variants.	9
Table S9	Association of S-gene detection with prior documented infections occurring before or after receipt of ≥2 vaccine doses	10
Table S10	Association of S-gene detection with immunocompromised or immunosuppressed status.	11
Table S11	Association of S-gene detection with prior documented infection among cases with differing COVID-19 vaccination status.	12
Table S12	Association of S-gene detection with prior vaccination with adjustment for natural infection.	13
Figure S1	Directed acyclic graph.	14

Table S1: History of vaccination and natural infection among cases with S-gene target failure or S-gene detection.

History of vaccination	History of recorded infections—count, *n* (%)							
	0 infections		1 infection		2 infections		3 infections	
	SGTF	S-gene detected	SGTF	S-gene detected	SGTF	S-gene detected	SGTF	S-gene detected
	N=15,212	*N*=6,377	*N*=6,180	*N*=3,181	*N*=462	*N*=303	*N*=16	*N*=8
0 doses	1,580 (10.4)	752 (11.8)	1,033 (16.7)	512 (16.1)	87 (18.8)	50 (16.5)	4 (25.0)	0
1 dose (any)	279 (1.8)	133 (2.1)	204 (3.3)	99 (3.1)	16 (3.5)	13 (4.3)	0	0
2 doses (any)	2,910 (19.1)	1,156 (18.1)	1,608 (26.0)	824 (25.9)	126 (27.3)	82 (27.1)	4 (25.0)	3 (37.5)
3 doses (any)	5,887 (38.7)	2,292 (35.9)	2,408 (39.0)	1,217 (38.3)	186 (40.3)	120 (39.6)	6 (37.5)	3 (37.5)
4 doses (any)	2,946 (19.4)	1,299 (20.4)	681 (11.0)	388 (12.2)	35 (7.6)	31 (10.2)	2 (12.5)	2 (25.0)
≥5 doses (any)	1,610 (10.6)	745 (11.7)	246 (4.0)	141 (4.4)	12 (2.6)	7 (2.3)	0	0

SGTF: S-gene target failure, defined as cycle threshold readings of >37 for the S-gene and ≤37 for N and orf1a/b genes.

Table S2: Association of S-gene detection with prior vaccination before or after natural SARS-CoV-2 infection.

Doses received, by timing in relation to individuals' first documented infection	Count, *n* (%)		Odds ratio (95% CI)		
	SGTF (Non-XBB lineage)	S-gene detected (XBB lineage)	Unadjusted	Time-adjusted[1]	Adjusted[2]
	N=21,870	*N*=9,869			
0 doses	2,704 (12.4)	1,314 (13.3)	ref.	ref.	ref.
0 dose before infection, ≥1 dose after infection	2,399 (11.0)	1,348 (13.7)	1.16 (1.05, 1.27)	1.15 (1.03, 1.27)	1.10 (0.97, 1.26)
1 dose before infection, 0 doses after infection	394 (1.8)	190 (1.9)	0.99 (0.82, 1.19)	0.97 (0.79, 1.18)	0.98 (0.80, 1.20)
1 dose before infection, ≥1 dose after infection	175 (0.8)	95 (1.0)	1.12 (0.86, 1.45)	1.00 (0.76, 1.33)	0.97 (0.73, 1.31)
2 doses before infection, 0 doses after infection	3,729 (17.1)	1,578 (16.0)	0.87 (0.80, 0.95)	0.86 (0.79, 0.95)	0.83 (0.74, 0.94)
2 doses before infection, ≥1 dose after infection	877 (4.0)	418 (4.2)	0.98 (0.86, 1.12)	0.93 (0.80, 1.07)	0.79 (0.66, 0.95)
3 doses before infection, 0 doses after infection	6,575 (30.1)	2,603 (26.4)	0.81 (0.75, 0.88)	0.86 (0.79, 0.93)	0.83 (0.74, 0.93)
3 doses before infection, ≥1 dose after infection	387 (1.8)	219 (2.2)	1.16 (0.97, 1.39)	0.98 (0.81, 1.19)	0.81 (0.65, 1.02)
4 doses before infection, 0 doses after infection	2,973 (13.6)	1,320 (13.4)	0.91 (0.83, 1.00)	0.91 (0.82, 1.00)	0.85 (0.75, 0.96)
4 doses before infection, ≥1 dose after infection	41 (0.2)	37 (0.4)	1.86 (1.18, 2.91)	1.33 (0.81, 2.17)	1.01 (0.60, 1.68)
≥5 doses before infection, 0 doses after infection	1,616 (7.4)	747 (7.6)	0.95 (0.85, 1.06)	0.83 (0.74, 0.94)	0.73 (0.63, 0.85)

SGTF: S-gene target failure, defined as cycle threshold readings of >37 for the S-gene and ≤37 for N and orf1a/b genes.

[1]Time-adjusted estimates are obtained via models defining intercepts for calendar week only. Unadjusted and time-adjusted estimates are not directly comparable across vaccination series due to differences in individuals' history of documented infection (e.g., any documented SARS-CoV-2 infection or no documented SARS-CoV-2 infection) within strata that received any doses or no doses prior to natural infection.

[2]Adjusted estimates are obtained via models adjusted for history of natural infection (1 or ≥2 infections before and after receipt of ≥2 COVID-19 vaccine doses), calendar week, age (10-year bands), sex, race/ethnicity, current or former cigarette smoking, body mass index, Charlson comorbidity index, neighborhood socioeconomic status, and prior-year healthcare utilization across outpatient, inpatient, and emergency department settings. Covariates are categorized as listed in **Table 1**.

Table S3: Association of S-gene detection with prior documented infection and COVID-19 vaccination, estimated via models defining continuous functions for calendar time.

Exposure	Primary analysis OR estimates		Alternative analysis OR estimates	
	Time-adjusted[1]	Adjusted[2]	Time-adjusted[1]	Adjusted[2]
Prior infection				
0 previous infections	ref.	ref.	ref.	ref.
1 previous infection	1.17 (1.10, 1.24)	1.17 (1.11, 1.24)	1.17 (1.10, 1.24)	1.17 (1.11, 1.24)
≥2 previous infections	1.39 (1.18, 1.63)	1.40 (1.19, 1.65)	1.39 (1.19, 1.63)	1.41 (1.20, 1.66)
COVID-19 vaccination[3]				
0 doses	ref.	ref.	ref.	ref.
1 dose (any)	1.01 (0.84, 1.21)	1.01 (0.84, 1.22)	1.01 (0.84, 1.20)	1.01 (0.84, 1.22)
2 doses (any)	0.91 (0.83, 1.00)	0.90 (0.82, 0.99)	0.91 (0.83, 1.00)	0.90 (0.82, 0.99)
3 doses (any)	0.91 (0.84, 0.99)	0.89 (0.81, 0.97)	0.91 (0.84, 0.98)	0.88 (0.81, 0.97)
4 doses (any)	0.94 (0.85, 1.03)	0.87 (0.79, 0.97)	0.93 (0.85, 1.02)	0.87 (0.79, 0.97)
≥5 doses (any)	0.85 (0.76, 0.95)	0.75 (0.66, 0.85)	0.85 (0.76, 0.95)	0.74 (0.65, 0.85)

SGTF: S-gene target failure, defined as cycle threshold readings of >37 for the S-gene and ≤37 for N and orf1a/b genes.
[1]Time-adjsuted estimates are obtained via models defining polynomial transformations of the calendar date of testing only.
[2]Adjusted estimates are obtained via models adjusted for polynomial transformations of the calendar date of testing, age (10-year bands), sex, race/ethnicity, current or former cigarette smoking, body mass index, Charlson comorbidity index, neighborhood socioeconomic status, and prior-year healthcare utilization across outpatient, inpatient, and emergency department settings. Covariates are categorized as listed in **Table 1**. Models including first, second, third, and fourth order polynomial terms for individuals' calendar date of testing were found to provide optimal penalized fit to data via the Bayesian Information Criterion.

Table S4: Association of S-gene detection with prior COVID-19 vaccination status, according to timing of most recent vaccination.

Prior vaccination	Count, *n* (%)		Odds ratio (95% CI)		
	SGTF (Non-XBB lineage)	S-gene detected (XBB lineage)	Unadjusted	Time-adjusted[1]	Adjusted[2]
	N=21,870	*N*=9,869			
By recent receipt of ≥1 dose					
0 doses	2,704 (12.4)	1,314 (13.3)	ref.	ref.	ref.
1 dose (0 within 90 days)	487 (2.2)	241 (2.4)	1.02 (0.86, 1.20)	1.01 (0.85, 1.22)	1.02 (0.85, 1.22)
1 dose (1 within 90 days)	12 (0.1)	4 (<0.1)	– –	– –	– –
2 dose (0 within 90 days)	4,612 (21.1)	2,051 (20.8)	0.92 (0.84, 0.99)	0.91 (0.83, 1.00)	0.90 (0.82, 0.99)
2 dose (≥1 within 90 days)	36 (0.2)	14 (0.1)	– –	– –	– –
3 dose (0 within 90 days)	8,302 (38.0)	3,553 (36.0)	0.80 (0.82, 0.95)	0.91 (0.83, 0.98)	0.88 (0.81, 0.97)
3 dose (≥1 within 90 days)	185 (0.8)	79 (0.8)	0.88 (0.67, 1.15)	0.99 (0.74, 1.32)	0.95 (0.71, 1.27)
≥4 dose (0 within 90 days)	3,934 (18.0)	2,002 (20.3)	1.05 (0.96, 1.14)	0.92 (0.84, 1.01)	0.83 (0.75, 0.92)
≥4 dose (≥1 within 90 days)	1,598 (7.3)	611 (6.2)	0.79 (0.70, 0.88)	0.86 (0.76, 0.97)	0.85 (0.75, 0.97)
Days from last dose receipt	Median (interquartile range)				
1-dose recipients	511 (408, 617)	553 (433, 640)	– –	– –	– –
2-dose recipients	515 (396, 599)	535 (416, 615)	– –	– –	– –
3-dose recipients	353 (317, 386)	365 (324, 401)	– –	– –	– –
≥4-dose recipients	84 (54, 153)	103 (64, 155)	– –	– –	– –

SGTF: S-gene target failure, defined as cycle threshold readings of >37 for the S-gene and ≤37 for N and orf1a/b genes.
[1]Time-adjsuted estimates are obtained via models defining intercepts for calendar week only.
[2]Adjusted estimates are obtained via models adjusted for calendar week, age (10-year bands), sex, race/ethnicity, current or former cigarette smoking, body mass index, Charlson comorbidity index, neighborhood socioeconomic status, and prior-year healthcare utilization across outpatient, inpatient, and emergency department settings. Covariates are categorized as listed in **Table 1**.

Table S5: Association of S-gene detection with prior COVID-19 vaccination status, according to vaccine type.

Prior vaccination	Count, *n* (%)		Odds ratio (95% CI)		
	SGTF (Non-XBB lineage)	S-gene detected (XBB lineage)	Unadjusted	Time-adjusted[1]	Adjusted[2]
	N=21,870	*N*=9,869			
By receipt of bivalent vaccine					
0 doses	2,704 (12.4)	1,314 (13.3)	ref.	ref.	ref.
1 dose (0 bivalent)	497 (2.3)	244 (2.5)	1.01 (0.85, 1.19)	1.01 (0.84, 1.21)	1.02 (0.85, 1.22)
1 dose (≥1 bivalent)	2 (<0.1)	1 (<0.1)	– –	– –	– –
2 dose (0 bivalent)	4,639 (21.2)	2,059 (20.9)	0.91 (0.84, 0.99)	0.91 (0.83, 1.00)	0.90 (0.82, 0.99)
2 dose (≥1 bivalent)	9 (<0.1)	6 (0.1)	– –	– –	– –
3 dose (0 bivalent)	8,285 (37.9)	3,517 (35.6)	0.87 (0.81, 0.94)	0.91 (0.83, 0.98)	0.89 (0.81, 0.97)
3 dose (≥1 bivalent)	202 (0.9)	115 (1.2)	1.17 (0.92, 1.49)	0.98 (0.76, 1.28)	0.95 (0.73, 1.23)
≥4 dose (0 bivalent)	1,788 (8.2)	765 (7.8)	0.88 (0.79, 0.98)	0.98 (0.88, 1.10)	0.87 (0.77, 0.99)
≥4 dose (≥1 bivalent)	3,744 (17.1)	1,848 (18.7)	1.02 (0.93, 1.11)	0.87 (0.80, 0.96)	0.82 (0.74, 0.91)

SGTF: S-gene target failure, defined as cycle threshold readings of >37 for the S-gene and ≤37 for N and orf1a/b genes.

[1]Time-adjsuted estimates are obtained via models defining intercepts for calendar week only.

[2]Adjusted estimates are obtained via models adjusted for calendar week, age (10-year bands), sex, race/ethnicity, current or former cigarette smoking, body mass index, Charlson comorbidity index, neighborhood socioeconomic status, and prior-year healthcare utilization across outpatient, inpatient, and emergency department settings. Covariates are categorized as listed in **Table 1**.

Table S6: Association of S-gene detection with most recent vaccine product received.

Last vaccine dose received	Count, *n* (%)		Odds ratio (95% CI)		
	SGTF (Non-XBB lineage)	S-gene detected (XBB lineage)	Unadjusted[1]	Adjusted for time and vaccine doses received[2]	Adjusted for all covariates, including vaccine doses received[3]
	N=21,870	*N*=9,869			
No history of vaccination	2,704 (12.4)	1,314 (13.3)	ref.	ref.	ref.
BNT162b2 monovalent	8,490	3,674	1.05 (0.85, 1.29)	1.04 (0.83, 1.30)	1.05 (0.84, 1.31)
BNT162b2 bivalent	2,394	1,151	1.42 (1.12, 1.79)	0.95 (0.74, 1.23)	0.98 (0.76, 1.27)
mRNA-1273 monovalent	6,266	2,703	1.05 (0.85, 1.29)	1.05 (0.84, 1.32)	1.06 (0.84, 1.33)
mRNA-1273 bivalent	1,562	817	1.53 (1.20, 1.95)	0.98 (0.76, 1.28)	1.01 (0.77, 1.31)
Ad.26.COV2.S	451	206	0.98 (0.81, 1.19)	0.99 (0.80, 1.21)	0.99 (0.80, 1.22)
NVX-CoV2373	2	2	– –	– –	– –

SGTF: S-gene target failure, defined as cycle threshold readings of >37 for the S-gene and ≤37 for N and orf1a/b genes.
[1]Unadjusted analyses do not control for differences in the number of vaccine doses received or
[2]Time-adjsuted estimates are obtained via models defining intercepts for calendar week as well as number of vaccine doses received.
[3]Adjusted estimates are obtained via models adjusted for number of vaccine doses received, calendar week, age (10-year bands), sex, race/ethnicity, current or former cigarette smoking, body mass index, Charlson comorbidity index, neighborhood socioeconomic status, and prior-year healthcare utilization across outpatient, inpatient, and emergency department settings.

Table S7: Vaccine dose sequences among cases with S-gene target failure or S-gene detection.

Total doses received	Sequence of doses	Count, *n* (%)	
		SGTF	S-gene detected
		N=21,870	*N*=9,869
1 dose			
	Ad.26.COV2.S	291 (1.3)	139 (1.4)
	BNT162b2 monovalent	142 (0.6)	67 (0.7
	mRNA-1273 monovalent	64 (0.3)	36 (0.4)
2 doses			
	BNT162b2 monovalent only	2,667 (12.2)	1,260 (12.8)
	mRNA-1273 monovalent only	1,504 (6.9)	616 (6.2)
	Ad.26.COV2.S, BNT162b2 monovalent	164 (0.7)	58 (0.6)
	Ad.26.COV2.S only	145 (0.7)	61 (0.6)
	Ad.26.COV2.S, mRNA-1273 monovalent	122 (0.6)	55 (0.6)
	BNT162b2 monovalent, mRNA-1273 monovalent	9 (<0.1)	3 (<0.1)
	mRNA-1273 monovalent, BNT162b2 monovalent	6 (<0.1)	1 (<0.1)
3 doses[1]			
	BNT162b2 monovalent only	4,303 (19.7)	1,828 (18.5)
	mRNA-1273 monovalent only	3,373 (15.4)	1,471 (14.9)
	mRNA-1273 monovalent (2), BNT162b2 monovalent	230 (1.1)	85 (0.9)
	BNT162b2 monovalent (2), mRNA-1273 monovalent	191 (0.9)	82 (0.8)
	BNT162b2 monovalent (2), BNT162b2 bivalent	90 (0.4)	46 (0.5)
	mRNA-1273 monovalent (2), mRNA-1273 bivalent	34 (0.2)	25 (0.3)
	Ad.26.COV2.S (2), BNT162b2 bivalent	19 (0.1)	16 (0.2)
	Ad.26.COV2.S, mRNA-1273 monovalent (2)	23 (0.1)	3 (<0.1)
	Ad.26.COV2.S, BNT162b2 monovalent, BNT162b2 bi20valent	20 (0.1)	10 (0.1)
	Ad.26.COV2.S (2), BNT162b2 monovalent	20 (0.1)	3 (<0.1)
	mRNA-1273 monovalent (2), BNT162b2 bivalent	13 (0.1)	8 (0.1)
	Ad.26.COV2.S (2), BNT162b2 monovalent	14 (0.1)	4 (<0.1)
4 doses[1]			
	BNT162b2 monovalent (3), BNT162b2 bivalent	923 (4.2)	462 (4.7)
	mRNA-1273 monovalent (4)	784 (3.6)	364 (3.7)
	BNT162b2 monovalent (4)	747 (3.4)	301 (3.0)
	mRNA-1273 monovalent (3), mRNA-1273 bivalent	652 (3.0)	331 (3.4)
	mRNA-1273 monovalent (3), BNT162b2 bivalent	217 (1.0)	97 (1.0)
	BNT162b2 monovalent (3), mRNA-1273 bivalent	63 (0.3)	29 (0.3)
	BNT162b2 monovalent (2), mRNA-1273 monovalent, BNT162b2 bivalent	41 (0.2)	19 (0.2)
	mRNA-1273 monovalent (2), BNT162b2 monovalent, BNT162b2 bivalent	37 (0.2)	23 (0.2)
	BNT162b2 monovalent (2), mRNA-1273 monovalent, mRNA-1273 bivalent	30 (0.1)	16 (0.2)
	mRNA-1273 monovalent (3), BNT162b2 monovalent	22 (0.1)	15 (0.2)
	BNT162b2 monovalent (3), mRNA-1273 monovalent	26 (0.1)	11 (0.1)
	mRNA-1273 monovalent (2), BNT162b2 monovalent (2)	23 (0.1)	9 (0.1)
≥5 doses[1]			
	BNT162b2 monovalent (4), BNT162b2 bivalent	786 (3.6)	356 (3.6)
	mRNA-1273 monovalent (4), mRNA-1273 bivalent	710 (3.2)	371 (3.8)
	mRNA-1273 monovalent (4), BNT162b2 bivalent	197 (0.9)	81 (0.8)
	BNT162b2 monovalent (3), mRNA-1273 monovalent, BNT162b2 bivalent	29 (0.1)	9 (0.1)
	BNT162b2 monovalent (4), mRNA-1273 bivalent	28 (0.1)	10 (0.1)
	BNT162b2 monovalent (5)	22 (0.1)	15 (0.2)
	mRNA-1273 monovalent (5)	21 (0.1)	17 (0.2)
	mRNA-1273 monovalent (3), BNT162b2 monovalent, BNT162b2 bivalent	26 (0.1)	8 (0.1)
	BNT162b2 monovalent (3), mRNA-1273 monovalent, mRNA-1273 bivalent	19 (0.1)	4 (<0.1)
	mRNA-1273 monovalent (3), BNT162b2 monovalent, mRNA-1273 bivalent	9 (<0.1)	5 (0.1)
	mRNA-1273 monovalent (2), BNT162b2 monovalent, mRNA-1273 monovalent, mRNA-1273 bivalent	9 (<0.1)	4 (<0.1)
	mRNA-1273 monovalent (2), BNT162b2 monovalent (2), BNT162b2 bivalent	9 (<0.1)	2 (<0.1)

SGTF: S-gene target failure, defined as cycle threshold readings of >37 for the S-gene and ≤37 for N and orf1a/b genes.
[1]The 12 most common sequences of 3, 4, and ≥5 doses are presented due to low counts for other dose histories.

Table S8: Association of S-gene detection with prior documented infections during periods with differing predominant circulating variants.

Prior infection	Count, *n* (%)		Odds ratio (95% CI)		
	SGTF (Non-XBB lineage)	S-gene detected (XBB lineage)	Unadjusted	Time-adjusted[1]	Adjusted[2]
	N=21,870	*N*=9,869			
0 documented infections	6,180 (28.3)	3,181 (32.2)	ref.	ref.	ref.
Prior documented infection during BA.4/BA.5 period[3]	333 (1.5)	316 (3.2)	2.14 (1.83, 2.50)	1.65 (1.39, 1.96)	1.67 (1.40, 1.99)
Prior documented infection during BA.1/BA.2 period[3]	3,292 (15.1)	1,565 (15.9)	1.06 (1.00, 1.14)	1.01 (0.94, 1.09)	1.04 (0.97, 1.12)
Prior documented infection during Delta[3]	802 (3.7)	413 (4.2)	1.15 (1.02, 1.30)	1.15 (1.01, 1.31)	1.18 (1.03, 1.35)
Prior documented infection during Alpha/Epsilon period[3]	770 (3.5)	416 (4.2)	1.21 (1.07, 1.36)	1.17 (1.03, 1.34)	1.19 (1.04, 1.36)
Prior documented infection during pre-Alpha/Epsilon period[3]	1,910 (8.7)	1,080 (10.9)	1.28 (1.19, 1.39)	1.27 (1.17, 1.39)	1.29 (1.18, 1.41)

SGTF: S-gene target failure, defined as cycle threshold readings of >37 for the S-gene and ≤37 for N and orf1a/b genes. We define periods as by date ranges during which the respective variants accounted for ≥50% of cases detected among all sequenced specimens within KPSC. These include: 1 January, 2020 to 3 January, 2021 (pre-Alpha/Epsilon period); 4 January, 2021 to 19 June, 2021 (Alpha/Epsilon period); 20 June, 2021 to 19 December, 2021 (Delta period); 20 December, 2021 to 24 June, 2022 (BA.1/BA.2 period); 25 June, 2022 to 30 November, 2022 (BA.4/BA.5 period).

[1]Time-adjsuted estimates are obtained via models defining intercepts for calendar week only.

[2]Adjusted estimates are obtained via models adjusted for calendar week, age (10-year bands), sex, race/ethnicity, current or former cigarette smoking, body mass index, Charlson comorbidity index, neighborhood socioeconomic status, and prior-year healthcare utilization across outpatient, inpatient, and emergency department settings. Covariates are categorized as listed in **Table 1**.

[3]Individuals with a documented infection during each period are included, who may have experienced or may not have experienced infections during other periods. Infections during each period are not mutually exclusive.

Table S9: Association of S-gene detection with prior documented infections occurring before or after receipt of ≥2 vaccine doses.

	Count, *n* (%)		Odds ratio (95% CI)		
	SGTF (Non-XBB lineage)	S-gene detected (XBB lineage)	Unadjusted	Time-adjusted[1]	Adjusted[2]
	N=21,870	*N*=9,869			
Infections before receipt of ≥2 vaccine doses					
0 documented infections	18,063 (82.6)	7,807 (79.1)	ref.	ref.	ref.
1 documented infection	3,660 (16.7)	1,970 (20.0)	1.27 (1.19, 1.35)	1.24 (1.16, 1.32)	1.22 (1.14, 1.31)
≥2 documented infections	147 (0.7)	92 (0.9)	1.49 (1.14, 1.93)	1.33 (1.00, 1.77)	1.30 (0.97, 1.73)
Any documented infection	3,807 (17.4)	2,062 (20.9)	1.27 (1.20, 1.35)	1.24 (1.16, 1.32)	1.23 (1.14, 1.31)
Infections after receipt of ≥2 vaccine doses					
0 documented infections	18,747 (85.7)	8,262 (83.7)	ref.	ref.	ref.
1 documented infection	3,052 (14.0)	1,559 (15.8)	1.19 (1.11, 1.27)	1.10 (1.02, 1.18)	1.13 (1.05, 1.22)
≥2 documented infections	71 (0.3)	48 (0.5)	1.58 (1.09, 2.28)	1.35 (0.90, 2.02)	1.39 (0.92, 2.09)
Any documented infection	3,123 (14.3)	1,607 (16.3)	1.20 (1.12, 1.28)	1.10 (1.03, 1.19)	1.13 (1.05, 1.22)

SGTF: S-gene target failure, defined as cycle threshold readings of >37 for the S-gene and ≤37 for N and orf1a/b genes.

[1]Time-adjsuted estimates are obtained via models defining intercepts for calendar week only.

[2]Adjusted estimates are obtained via models adjusted for calendar week, age (10-year bands), sex, race/ethnicity, current or former cigarette smoking, body mass index, Charlson comorbidity index, neighborhood socioeconomic status, and prior-year healthcare utilization across outpatient, inpatient, and emergency department settings. Covariates are categorized as listed in **Table 1**.

Table S10: Association of S-gene detection with immunocompromised or immunosuppressed status.

	Count, *n* (%)		Odds ratio (95% CI)		
	SGTF (Non-XBB lineage)	S-gene detected (XBB lineage)	Unadjusted	Time-adjusted[1]	Adjusted[2]
All cases	*N*=21,870	*N*=9,869			
Solid organ transplant	58 (0.3)	21 (0.2)	0.86 (0.57, 1.33)	0.71 (0.40, 1.23)	0.79 (0.43, 1.05)
Cancer	677 (3.1)	298 (3.0)	1.03 (0.90, 1.18)	0.99 (0.88, 1.13)	0.88 (0.82, 1.06)
HIV infection	22 (0.1)	19 (0.2)	2.10 (1.26, 3.57)	2.03 (1.01, 3.98)	2.31 (1.56, 2.86)
Rheumatic disorders	317 (1.4)	136 (1.4)	0.98 (0.83, 1.17)	0.97 (0.82, 1.15)	0.92 (0.80, 1.10)
Other immunocompromise or immunosuppression	852 (3.9)	380 (3.9)	1.01 (0.91, 1.12)	1.01 (0.87, 1.06)	0.90 (0.81, 1.00)
Any immunocompromise or immunosuppression	1,723 (7.9)	766 (7.8)	1.00 (0.93, 1.08)	0.95 (0.88, 1.01)	0.95 (0.89, 1.05)

SGTF: S-gene target failure, defined as cycle threshold readings of >37 for the S-gene and ≤37 for N and orf1a/b genes.
[1]Time-adjsuted estimates are obtained via models defining intercepts for calendar week only.
[2]Adjusted estimates are obtained via models adjusted for calendar week, age (10-year bands), sex, race/ethnicity, current or former cigarette smoking, body mass index, Charlson comorbidity index, neighborhood socioeconomic status, and prior-year healthcare utilization across outpatient, inpatient, and emergency department settings. Covariates are categorized as listed in **Table 1**.

Table S11: Association of S-gene detection with prior documented infection among cases with differing COVID-19 vaccination status.

Exposure	Count, *n* (%)		Odds ratio (95% CI)		
	SGTF (Non-XBB lineage)	S-gene detected (XBB lineage)	Unadjusted	Time-adjusted[1]	Adjusted[2]
	N=21,870	*N*=9,869			
0 vaccine doses					
0 previous infections	1,580	752	ref.	ref.	ref.
1 previous infection	1,033	512	1.04 (0.91, 1.19)	0.98 (0.85, 1.14)	0.99 (0.85, 1.15)
≥2 previous infections	91	50	1.15 (0.81, 1.65)	1.01 (0.68, 1.48)	1.02 (0.69, 1.51)
1 vaccine dose					
0 previous infections	279	133	ref.	ref.	ref.
1 previous infection	204	99	1.02 (0.74, 1.40)	1.03 (0.73, 1.45)	1.04 (0.74, 1.46)
≥2 previous infections	16	13	1.70 (0.79, 3.62)	1.45 (0.63, 3.33)	1.45 (0.63, 3.34)
2 vaccine doses					
0 previous infections	2,910	1,156	ref.	ref.	ref.
1 previous infection	1,608	824	1.29 (1.16, 1.43)	1.28 (1.14, 1.44)	1.29 (1.15, 1.45)
≥2 previous infections	130	85	1.65 (1.24, 2.18)	1.52 (1.12, 2.05)	1.52 (1.12, 2.07)
3 vaccine doses					
0 previous infections	5,887	2,292	ref.	ref.	ref.
1 previous infection	2,408	1,217	1.30 (1.19, 1.41)	1.19 (1.08, 1.30)	1.19 (1.09, 1.30)
≥2 previous infections	192	123	1.65 (1.31, 2.08)	1.40 (1.09, 1.80)	1.42 (1.10, 1.83)
≥4 vaccine doses					
0 previous infections	4,556	2,044	ref.	ref.	ref.
1 previous infection	927	529	1.27 (1.13, 1.43)	1.16 (1.02, 1.32)	1.17 (1.02, 1.33)
≥2 previous infections	49	40	1.82 (1.19, 2.78)	1.68 (1.06, 2.67)	1.73 (1.08, 2.74)

SGTF: S-gene target failure, defined as cycle threshold readings of >37 for the S-gene and ≤37 for N and orf1a/b genes.
[1]Time-adjsuted estimates are obtained via models defining intercepts for calendar week only.
[2]Adjusted estimates are obtained via models adjusted for calendar week, age (10-year bands), sex, race/ethnicity, current or former cigarette smoking, body mass index, Charlson comorbidity index, neighborhood socioeconomic status, and prior-year healthcare utilization across outpatient, inpatient, and emergency department settings. Covariates are categorized as listed in **Table 1**.

Table S12: Association of S-gene detection with prior vaccination with adjustment for natural infection.

COVID-19 vaccine doses received	Count, *n* (%)		Adjusted odds ratio
	SGTF (Non-XBB lineage)	S-gene detected (XBB lineage)	Est. (95% CI)[2]
	N=21,870	*N*=9,869	
0 doses	2,704 (12.4)	1,314 (13.3)	ref.
1 dose (any)	499 (2.3)	245 (2.5)	1.02 (0.85, 1.22)
2 doses (any)	4,648 (4,648)	2,065 (20.9)	0.91 (0.83, 1.00)
3 doses (any)	8,487 (8,487)	3,632 (36.8)	0.91 (0.83, 0.99)
4 doses (any)	3,664 (3,664)	1,720 (17.4)	0.91 (0.82, 1.01)
≥5 doses (any)	1,868 (1,868)	893 (9.0)	0.78 (0.69, 0.89)

SGTF: S-gene target failure, defined as cycle threshold readings of >37 for the S-gene and ≤37 for N and orf1a/b genes.
[1]Time-adjsuted estimates are obtained via models defining intercepts for calendar week only.
[2]Adjusted estimates are obtained via models adjusted for calendar week, age (10-year bands), sex, race/ethnicity, current or former cigarette smoking, body mass index, Charlson comorbidity index, neighborhood socioeconomic status, and prior-year healthcare utilization across outpatient, inpatient, and emergency department settings, and prior vaccination or prior infection, as applicable. Covariates are categorized as listed in **Table 1**.

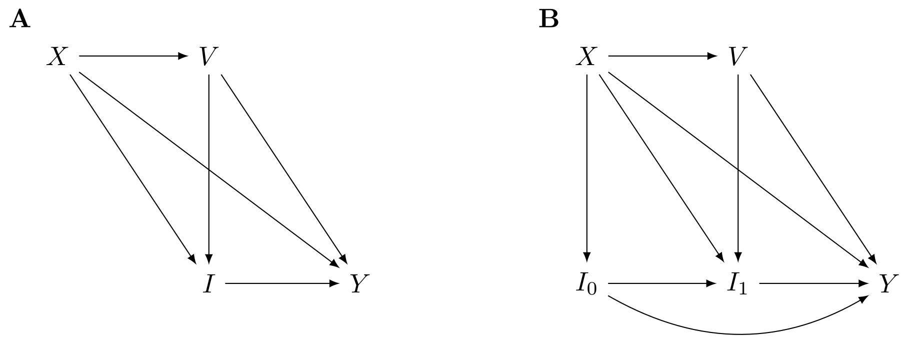

Figure S1: Directed acyclic graphs. We illustrate two directed acyclic graphs motivating the analysis frameworks employed. In the left panel (**A**), V denotes individuals' COVID-19 vaccination status (including number and timing of doses); I denotes individuals' prior infection history; Y denotes individuals' outcome of detected infection with a particular lineage; and X denotes all other determinants of individuals' risk of detected infection with a particular lineage, which may include demographic factors, comorbid conditions influencing susceptibility, and healthcare-seeking behavior. Here, we expect that X may influence individuals' likelihood of receiving vaccination, as well as their risk of infection historically ($X \rightarrow I$) and at present ($X \rightarrow Y$). Estimating the total effect of V on Y requires adjustment for X, while estimating the total effect of I on Y requires adjustment for both V and X. Estimating the direct effect of V on Y, i.e. the independent effect of vaccination on risk of acquiring a particular strain (not mediated by effects of prior vaccination on the individual's infection history) requires adjustment for I and X; when applying this analysis approach, we identify results nearly identical to total effect estimates (**Table S7**), suggesting that the observed relationship of V with Y is not substantially mediated by effects of V on I. Note that our use of the terms "direct" and "total" effect correspond with their interpretation in mediation analysis for vaccination as an individual-level exposure,[54] and are distinct from interpretation of the same terms in vaccine field trials.[55] In the right panel (**B**), we subdivide infection history into infections occurring before (I_0) or after (I_1) receipt of vaccination, corresponding to the estimates presented in **Table S4**. Here, estimating the total effect of V on Y requires adjustment only for X, while estimating the direct effect of V on Y requires adjustment for both I_0 and I_1 (corresponding to the approach taken in the **Table S4** analyses). Estimating the total effect of I_0 on Y requires adjustment for X only, and estimating the total effect of I_1 on Y requires adjustment for X, V, and I_0. Estimating the direct effects of I_0 and I_1 on Y requires adjustment for I_1 and I_0, respectively, as well as X and V (in both instances, corresponding to the approach taken in the **Table S4** analyses). While panels **A** and **B** treat vaccination status as a unitary variable, it remains important to consider that V may also comprise doses received before or after natural infection; in this event, I_0 may be considered to impact vaccination behaviors following infection. Analyses presented in **Table S1**, accordingly, disaggregate associations of infecting lineage with vaccine doses received either before or after documented natural infection.
