# Supplementary material for: Increased vaccine sensitivity of an emerging SARS-CoV-2 variant
Source: Nat Commun. 2023 Jun 29;14:3854. doi: 10.1038/s41467-023-39567-2 (PMC10310822; doi:10.1038/s41467-023-39567-2)
Supplement: Supplementary file 3 — Reporting Summary [file 41467_2023_39567_MOESM3_ESM.pdf]

nature portfolio

Corresponding author(s): Lewnard, Joseph

Last updated by author(s): Jun 14, 2023

Reporting Summary

Nature Portfolio wishes to improve the reproducibility of the work that we publish. This form provides structure for consistency and transparency in reporting. For further information on Nature Portfolio policies, see our Editorial Policies and the Editorial Policy Checklist.

Statistics

For all statistical analyses, confirm that the following items are present in the figure legend, table legend, main text, or Methods section.

n/a	Confirmed	
☐	☒	The exact sample size (*n*) for each experimental group/condition, given as a discrete number and unit of measurement
☐	☒	A statement on whether measurements were taken from distinct samples or whether the same sample was measured repeatedly
☒	☐	The statistical test(s) used AND whether they are one- or two-sided<br>*Only common tests should be described solely by name; describe more complex techniques in the Methods section.*
☐	☒	A description of all covariates tested
☐	☒	A description of any assumptions or corrections, such as tests of normality and adjustment for multiple comparisons
☐	☒	A full description of the statistical parameters including central tendency (e.g. means) or other basic estimates (e.g. regression coefficient) AND variation (e.g. standard deviation) or associated estimates of uncertainty (e.g. confidence intervals)
☒	☐	For null hypothesis testing, the test statistic (e.g. *F*, *t*, *r*) with confidence intervals, effect sizes, degrees of freedom and *P* value noted<br>*Give P values as exact values whenever suitable.*
☒	☐	For Bayesian analysis, information on the choice of priors and Markov chain Monte Carlo settings
☒	☐	For hierarchical and complex designs, identification of the appropriate level for tests and full reporting of outcomes
☒	☐	Estimates of effect sizes (e.g. Cohen's *d*, Pearson's *r*), indicating how they were calculated

Our web collection on statistics for biologists contains articles on many of the points above.

Software and code

Policy information about availability of computer code

Data collection	No software was used for data collection.
Data analysis	We conducted analyses using R (version 4.0.3; R Foundation for Statistical Computing, Vienna, Austria). We used the survival package (version 3.5-3) for time-to-event analyses, and the Amelia II package (version 1.81.1) for multiple imputation of missing data. Analysis code is available from GitHub (https://github.com/joelewnard/xbb).

For manuscripts utilizing custom algorithms or software that are central to the research but not yet described in published literature, software must be made available to editors and reviewers. We strongly encourage code deposition in a community repository (e.g. GitHub). See the Nature Portfolio guidelines for submitting code & software for further information.

Data

Policy information about availability of data

All manuscripts must include a data availability statement. This statement should provide the following information, where applicable:

- Accession codes, unique identifiers, or web links for publicly available datasets
- A description of any restrictions on data availability
- For clinical datasets or third party data, please ensure that the statement adheres to our policy

Individual-level testing and clinical outcomes data reported in this study are not publicly shared due to privacy protections for patient electronic health records. Individuals wishing to access disaggregated data, including data reported in this study, should submit requests for access to the corresponding author

(sara.y.tartof@kp.org). Requests will receive a response within 14 days. De-identified data (including, as applicable, participant data and relevant data dictionaries) will be shared upon approval of analysis proposals with signed data-access agreements in place.

Human research participants

Policy information about studies involving human research participants and Sex and Gender in Research.

Reporting on sex and gender	We analyze sex as a biological variable. Data on cases' biological sex are presented in Table 1.
Population characteristics	Age, sex, race, socioeconomic status (measured at the community level), smoking behavior, body mass index, comorbidities, and healthcare utilization variables within the population are tabulated in Table 1.
Recruitment	This analysis included all individuals who are members of Kaiser Permanente Southern California health plans (who had been continuously enrolled for ≥1 year at the time of the study, to support evaluation of comorbidities and healthcare utilization) who received a positive molecular diagnostic test for SARS-CoV-2 during the study period. Individuals in this insured cohort may have better healthcare access than those without commercial insurance. Additionally, the decision to seek outpatient SARS-CoV-2 testing may differ with other parameters of healthcare utilization within the population enrolled in KPSC health plans. Individuals who sought testing in clinical settings during this time, when home antigen testing was widely available, may not be representative of all individuals who acquired SARS-CoV-2 infection, leading to selection bias. However, we are unaware of reasons that such bias would be differential among cases infected with XBB and non-XBB lineages.
Ethics oversight	The study protocol was approved by the KPSC Institutional Review Board.

Note that full information on the approval of the study protocol must also be provided in the manuscript.

Field-specific reporting

Please select the one below that is the best fit for your research. If you are not sure, read the appropriate sections before making your selection.

☒ Life sciences ☐ Behavioural & social sciences ☐ Ecological, evolutionary & environmental sciences

For a reference copy of the document with all sections, see nature.com/documents/nr-reporting-summary-flat.pdf

Life sciences study design

All studies must disclose on these points even when the disclosure is negative.

Sample size	No predetermined sample size was specified for this observational study; all cases meeting eligibility criteria were included in analyses. The research team did not actively enroll or administer interventions to the study population. The available sample of 21,870 cases with SGTF and 9,869 cases with S-gene detection was determined to be sufficient for identifying a prevalence ratio ≥1.1 with ≥80% power for either the vaccination or prior infection exposures at two-sided p<0.05 significance threshold.
Data exclusions	We restricted our analytic sample to individuals who first tested positive in an outpatient setting to select on healthcare-seeking behavior within the study population, thus maximizing internal validity when comparing outcomes among XBB and non-XBB cases. We also restricted to cases whose tests were processed using the ThermoFisher TaqPath COVID-19 Combo Kit to provide S-gene detection readout for all cases. We excluded cases without 1 year of continuous enrollment to ensure complete covariate data were available. We excluded cases with a prior positive test result within 90 days to ensure our analyses addressed incident infections.
Replication	We undertook sensitivity and secondary analyses stratified by vaccination type and timing (Table S2), by prior infection timing (Table S3), by timing of prior infection in relation to vaccination (Table S4), by immunocompromised or immunosuppressed status (Table S5), and by the joint distribution of prior infection and vaccination (Table S6). These analyses confirmed the findings of primary analyses.<br><br>Additionally, as our analysis included multiple imputation of missing observations, all analyses were repeated across 5 pseudo-datasets with missing observations drawn from their conditional distributions with respect to other observed variables. Findings were consistent across all the individual imputed datasets. Reported results are pooled across these analyses.
Randomization	Our study was not randomized. Analyses controlled for the following characteristics for each case via covariate adjustment: age (defined in 10-year age bands), sex, race/ ethnicity (white, black, Hispanic of any race, Asian, Pacific Islander, and other/mixed/unknown race), neighborhood deprivation index, measured at the Census block level; smoking status (current, former, or never smoker); body mass index (BMI; underweight, normal weight, overweight, obese, and morbidly obese); Charlson comorbidity index (0, 1-2, 3-5, and ≥6); prior-year emergency department visits and inpatient admissions; and prior-year outpatient visits. Regression models defined strata according to cases' dates of testing to control for variation over time in healthcare seeking behavior and/or clinical practices.
Blinding	While the study was not strictly blinded, determinations of S gene target failure (proxy for XBB or non-XBB infection) were not included in patients' clinical record; thus, clinical personnel were unaware of whether patients' infecting lineage. As infecting lineage does not inform clinical management, such data were not considered relevant to clinical care provision. Furthermore, analyses linking S gene target failure results to patients' clinical data were undertaken retrospectively, after clinical follow-up of patients had already been completed. Data analysts were not blinded to cases' status of S gene target failure or S gene detection. Blinding of data analysts was not considered feasible due to the need for direct correspondence with clinical laboratory personnel, including the data management team.

Reporting for specific materials, systems and methods

We require information from authors about some types of materials, experimental systems and methods used in many studies. Here, indicate whether each material, system or method listed is relevant to your study. If you are not sure if a list item applies to your research, read the appropriate section before selecting a response.

Materials & experimental systems

n/a	Involved in the study
☒	☐ Antibodies
☒	☐ Eukaryotic cell lines
☒	☐ Palaeontology and archaeology
☒	☐ Animals and other organisms
☒	☐ Clinical data
☒	☐ Dual use research of concern

Methods

n/a	Involved in the study
☒	☐ ChIP-seq
☒	☐ Flow cytometry
☒	☐ MRI-based neuroimaging
